# Beyond Cartilage Regeneration: Targeting the Subchondral Bone with Extracorporeal Shock Wave Therapy and Regenerative Medicine in Knee Osteoarthritis

**DOI:** 10.3390/bioengineering13091046

**Published:** 2026-09-09

**Authors:** Shinya Nakasato, Koji Aso, Tsukasa Kumai

**Affiliations:** 1 Medical Corporation N Clinic, Kishiwada City 596-0045, Osaka, Japan; 2 Medical Corporation Honmachi N Clinic, Osaka City 550-0005, Osaka, Japan; 3 Graduate School of Sport Sciences, Waseda University, Tokorozawa City 359-1192, Saitama, Japan; 4 Department of Orthopedic Surgery, Kochi Medical School, Kochi University, Nankoku City 783-8505, Kochi, Japan; 5 Faculty of Sport Sciences, Waseda University, Tokorozawa City 359-1192, Saitama, Japan

**Keywords:** knee osteoarthritis, subchondral bone, bone marrow lesion, extracorporeal shock wave therapy, regenerative medicine, hard tissue engineering

## Abstract

Knee osteoarthritis (OA) has long been viewed as a cartilage-centered disease, but a whole-joint model now positions the subchondral bone and the osteochondral unit as biologically active, mechanically decisive participants. Bone marrow lesions (BMLs), abnormal subchondral remodeling, and progressive deterioration of the subchondral bone plate (SBP) precede cartilage failure, suggesting a potential pre-collapse therapeutic window before irreversible articular surface collapse. We review evidence for combining extracorporeal shock wave therapy (ESWT), which provides mechanotransductive conditioning of the subchondral compartment, with regenerative therapies—platelet-rich plasma (PRP), autologous protein solution (APS), mesenchymal stromal cells (MSCs), and bone marrow aspirate concentrate (BMAC)—delivered intra-articularly and intra-osseously. Two clinical messages emerge: (i) BMLs are initially reversible but may progress to SBP discontinuity, subchondral cysts, and articular surface collapse if untreated, so subchondral-directed therapy should ideally be delivered before SBP discontinuity and cyst formation become imaging-overt, and (ii) advanced radiographic disease, including Kellgren–Lawrence grade 4, does not by itself mandate arthroplasty, because intra-osseous orthobiologics with ESWT may—as a hypothesis requiring prospective validation—preserve the joint and defer surgery in selected patients. This hypothesis-generating framework positions the osteochondral unit as a tractable target for hard-tissue engineering and requires prospective validation.

## 1. Introduction

For much of the twentieth century, knee osteoarthritis (OA) was conceptualized as a disease of cartilage wear. Radiographic joint space narrowing was treated as its principal signature, and therapeutic development was oriented predominantly toward chondroprotection or cartilage regeneration. Yet decades of clinical trials of chondromodulating agents and cartilage-focused biologics have delivered inconsistent structural and symptomatic benefits, and no disease-modifying OA drug (DMOAD) has achieved regulatory approval for the knee to date [1,2]. Successive attempts at pharmacological cartilage modification—including chondroitin sulfate combinations, matrix metalloproteinase inhibitors, cathepsin K inhibitors, inducible nitric oxide synthase (iNOS) inhibitors, and, more recently, intra-articular fibroblast growth factor 18 (sprifermin) and Wnt pathway modulators—have generally failed to translate promising preclinical or radiographic signals into consistent, clinically meaningful long-term benefit [1,2]. Cell-based cartilage restoration procedures such as autologous chondrocyte implantation and matrix-associated approaches, although useful for focal chondral defects in relatively young patients, have not been shown to modify the natural history of established OA, particularly in the setting of subchondral involvement [1,2,3]. This cumulative experience has motivated a critical re-examination of the assumption that cartilage repair alone can arrest or reverse the disease trajectory of knee OA, and has renewed interest in the role of subchondral bone and the wider osteochondral unit as therapeutic targets.

**Figure 1:**
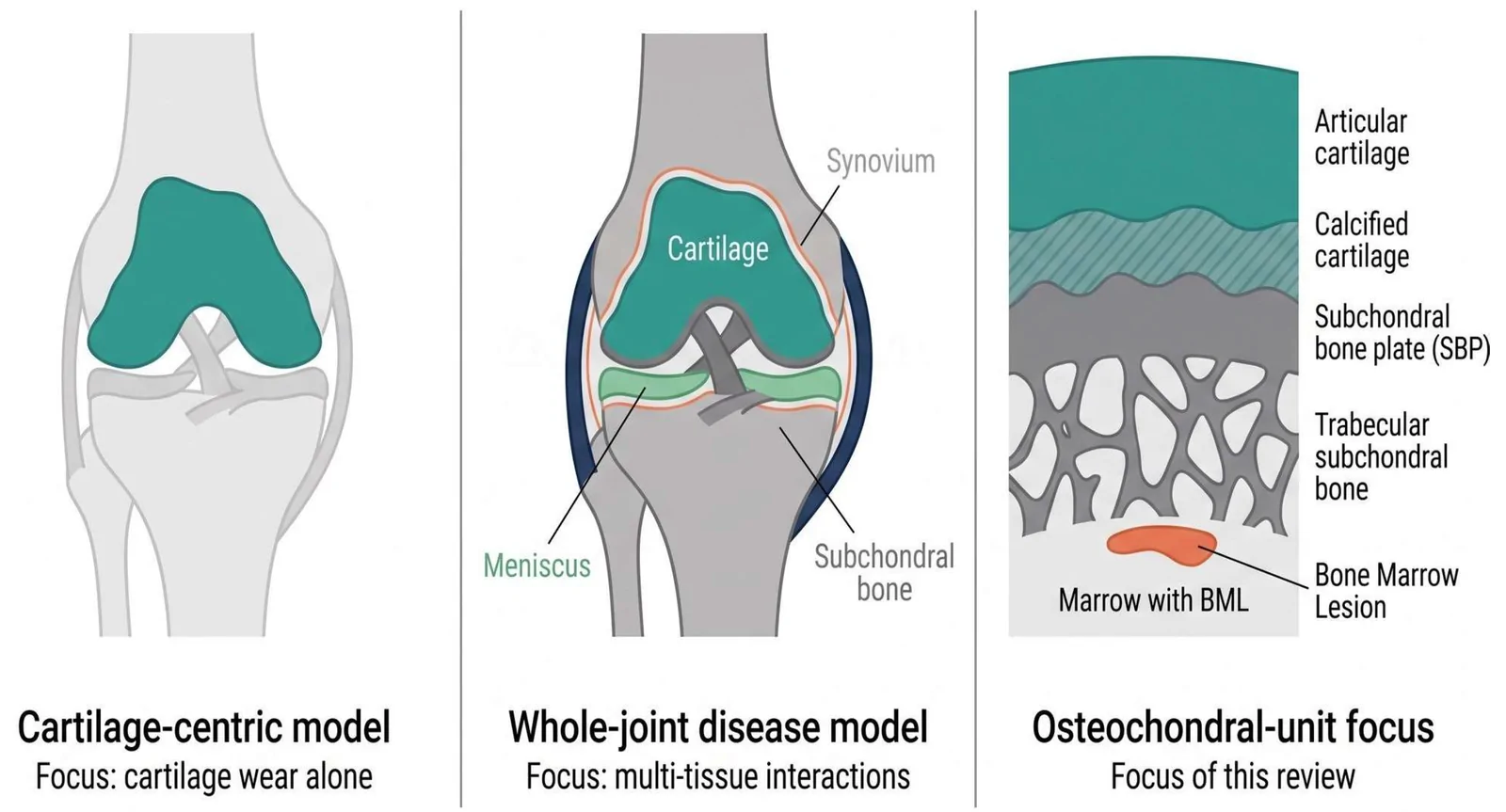
Evolution of the conceptual model of knee osteoarthritis. (**Left**) The historical cartilage-centric model, focused on cartilage wear alone. (**Middle**) The whole-joint disease model, recognizing cartilage, meniscus, synovium, ligaments, and subchondral bone as interacting contributors. (**Right**) The osteochondral-unit focus adopted in this review, comprising articular cartilage, calcified cartilage, the subchondral bone plate (SBP), trabecular subchondral bone, and marrow containing a bone marrow lesion (BML). Abbreviations: SBP, subchondral bone plate; BML, bone marrow lesion.

At the same time, a substantial body of imaging, histological, and epidemiological evidence has reframed knee OA as a whole-joint disorder involving cartilage, meniscus, synovium, ligaments, capsule, muscle, and—critically—subchondral bone [1,2] (Figure 1). Bone marrow lesions (BMLs), defined as ill-defined hyperintense regions on fluid-sensitive magnetic resonance imaging (MRI), are present in the majority of symptomatic patients and are two- to five-fold more common in painful than in non-painful knees; larger and progressing BMLs predict both incident radiographic OA and structural progression [4,5,6]. Histopathologically, symptomatic knee OA is associated with subchondral changes—including angiogenesis, osteoclastic remodeling, and nerve growth factor (NGF)-expressing osteochondral channels—that are independent of chondropathy and synovitis [7,8,9]. Biomechanically, the subchondral bone plate (SBP) and adjacent trabecular bone determine how load is transferred to overlying cartilage, and their stiffening or focal disruption alters the mechanical microenvironment in which chondrocytes and mesenchymal progenitors operate [3,10,11]. In parallel, longitudinal cohort studies from the Osteoarthritis Initiative and independent centers have shown that BML incidence, persistence, and volumetric progression track closely with radiographic and symptomatic worsening, positioning the subchondral compartment as a leading rather than lagging indicator of joint failure [4,5,6,12]. Epidemiological work has additionally linked BMLs to increased risk of total knee arthroplasty over follow-up periods of several years, further reinforcing their prognostic significance [4,5,12]. Together, these observations suggest that the subchondral compartment is not a passive substrate but an active driver of pain, structural progression, and treatment response.

From a hard-tissue bioengineering standpoint, this reframing has direct therapeutic consequences. If the subchondral bone is biologically active, mechanically decisive, and clinically relevant, then it should also be considered a legitimate target for engineered biological and mechanical interventions. The osteochondral unit—the graded composite of hyaline cartilage, calcified cartilage, SBP, and trabecular subchondral bone—is not simply a scaffold beneath cartilage; it is a dynamic, load-bearing biological system whose regeneration requires simultaneous attention to cells, signals, and the mechanical environment [13,14]. Contemporary hard-tissue engineering has increasingly recognized this graded architecture as a design constraint for osteochondral repair, motivating biphasic scaffolds, gradient bioinks, and combined mechanical–biological conditioning strategies [15,16,17]. Advances in 3D bioprinting and biofabrication now allow the construction of osteochondral analogues with region-specific mechanical, biochemical, and cellular properties, underscoring the importance of matching regenerative interventions to the underlying tissue architecture [16,17]. Regenerative approaches that ignore the mechanical and vascular state of the subchondral compartment may therefore underperform, irrespective of the sophistication of the biological product delivered.

Building on this perspective, we and other groups have argued that extracorporeal shock wave therapy (ESWT) and orthobiologic interventions—each individually supported by clinical and preclinical evidence—should be evaluated not as competing tools but as complementary components of a stage-appropriate strategy for the osteochondral unit. Focused ESWT is a non-invasive mechanical actuator that engages mechanotransductive pathways in bone, cartilage, and mesenchymal cells, whereas platelet-rich plasma (PRP), autologous protein solution (APS), and mesenchymal stromal cell (MSC)-based therapies provide biochemical and cellular signals that shape local repair. Their combination, in principle, allows the mechanical and biological environments of the subchondral compartment to be treated simultaneously.

This narrative review develops that argument in four steps. First, we summarize the biological and mechanical case for the subchondral bone as a key player in knee OA. Second, we propose that structural deterioration of the SBP—from an intact plate, to a disrupted-but-not-collapsed plate, to an overtly collapsed articular surface—represents a clinically meaningful spectrum, with important implications for treatment selection and expectation. Third, we examine how ESWT and regenerative medicine (PRP, APS, and MSCs) act as complementary modalities on this spectrum. Fourth, drawing on our own recent clinical observations [18], we outline a stage-dependent concept in which mechanical conditioning and biological regeneration are combined and prioritized before irreversible structural failure. Because much of the evidence linking these strands is preclinical, translational, or observational, all clinical claims are framed cautiously, and hypotheses are clearly distinguished from established evidence. The overall aim is to provide a conceptual scaffold that can guide the design of future prospective studies at the intersection of orthopedics, regenerative medicine, and hard-tissue bioengineering. Two overarching clinical messages are threaded through this review. The first is preventive: BMLs are potentially reversible in an early phase but progress toward irreversible subchondral structural change and, eventually, articular surface collapse if that phase is missed—so intervention should be prioritized before SBP discontinuity and subchondral cyst formation become imaging-overt and the lesion becomes irreversible. The second is restorative: advanced radiographic OA, including Kellgren–Lawrence (KL) grade 4 disease, is not by itself an unconditional indication for arthroplasty; the combination of intra-osseous orthobiologics with ESWT—understood as the two acting together, not orthobiologics alone—may, in selected patients—as a hypothesis requiring prospective validation—achieve joint preservation and defer or, in some cases, avoid surgery.

This article is a narrative (non-systematic) review. The literature was identified through searches of PubMed/MEDLINE, Scopus, and Web of Science from database inception to July 2026, using combinations of the terms “knee osteoarthritis”, “subchondral bone”, “subchondral bone plate”, “bone marrow lesion”, “osteochondral unit”, “extracorporeal shock wave therapy”, “platelet-rich plasma”, “autologous protein solution”, “mesenchymal stromal cells”, “bone marrow aspirate concentrate”, “intraosseous injection”, and “tissue engineering”, supplemented by manual screening of the reference lists of retrieved articles. We prioritized randomized controlled trials, systematic reviews and meta-analyses, and mechanistically informative preclinical studies published in English in peer-reviewed journals; case reports without imaging correlation were excluded. Because study selection followed the authors’ judgment of relevance to the conceptual framework rather than a pre-registered protocol, no formal risk-of-bias assessment was performed; this limitation is discussed in Section 9.

## 2. The Osteochondral Unit and Subchondral Bone in Knee OA

### 2.1. Anatomical and Biomechanical Overview

The osteochondral unit comprises articular hyaline cartilage, the calcified cartilage layer, the SBP (a thin cortical lamella typically 100–500 μm thick in adult human knees), and the underlying trabecular subchondral bone with its marrow spaces [13,14]. Functionally, these tissues form a graded mechanical continuum: the compliant hyaline cartilage distributes joint contact stress across the calcified cartilage into the stiffer SBP and, ultimately, into the trabecular network. The tidemark—the transition between uncalcified and calcified cartilage—together with the cement line at the calcified cartilage–SBP interface, functions as a graded mechanical bridge; duplication or advancement of the tidemark is a characteristic histological feature of OA and reflects reactivation of endochondral processes at the osteochondral interface [13,14,19]. The stiffness gradient across these layers—compliant uncalcified cartilage, intermediate calcified cartilage, stiff cortical SBP, and heterogeneous trabecular bone beneath—is essential for smooth stress transfer, and any local perturbation of this gradient concentrates stress at the interface and increases the risk of matrix failure [3,10,11].

A classic work by Radin and colleagues demonstrated that repetitive impulsive loading stiffens subchondral bone and increases stress at the cartilage–bone interface, predisposing to overlying cartilage failure [10]. Modern reviews reinforce that the mechanical properties of the subchondral compartment actively modulate cartilage strain, chondrocyte biology, and the propagation of joint injury [3,11]. Micro-computed tomography and high-resolution imaging studies have shown that OA-associated stiffening of the SBP is accompanied by a paradoxical increase in intracortical porosity and vascular channel density, producing a compartment that is at once mechanically more rigid and biologically more permeable to marrow-derived signals [3,11,20]. In advanced disease, malalignment—particularly varus deformity—shifts load toward the medial compartment, driving asymmetric SBP thickening, medial BML formation, and eventual medial articular surface collapse; the same principle applies with reversed geometry to valgus knees [3,10,11,12,19]. In tissue-engineering terms, the subchondral compartment functions as a living, remodelable substrate whose stiffness, porosity, and vascularity co-determine the fate of any overlying cartilage repair [15,16,17]. This provides a mechanical rationale for interventions that seek to modify the subchondral environment rather than only the articular surface.

### 2.2. Biological Cross-Talk

Cartilage and subchondral bone are separated by only a thin calcified interface. Vascular channels penetrating the SBP allow bidirectional diffusion of cytokines, growth factors, and matrix fragments, and immunohistochemical studies have documented increased density of these osteochondral channels in painful OA [7,8,20]. Signaling axes include transforming growth factor-β (TGF-β), bone morphogenetic proteins (BMPs), Wnt/β-catenin, receptor activator of nuclear factor-κB ligand/osteoprotegerin (RANKL/OPG), matrix metalloproteinases (MMPs), and vascular endothelial growth factor (VEGF) [3,11,21,22]. Excess TGF-β activity in subchondral bone, for example, has been linked in preclinical models to aberrant subchondral bone formation, uncoupled osteoclast–osteoblast remodeling, and cartilage degeneration, and pharmacological attenuation of TGF-β signaling in subchondral bone can delay experimental OA progression [21]. Wnt/β-catenin signaling similarly governs osteoblast differentiation and mineralization within the subchondral compartment; its dysregulation contributes both to sclerosis of the SBP and to abnormal turnover in the adjacent trabecular network [22]. BMP signaling, particularly BMP-2 and BMP-7, drives osteogenic differentiation and is one of the pathways engaged by mechanotransductive stimuli including ESWT [23,24]. VEGF-driven angiogenesis, in turn, brings nerves alongside new vessels into osteochondral channels, mechanistically linking remodeling with pain generation [7,8,9,20]. RANKL/OPG balance regulates osteoclast activity in the subchondral compartment and is shifted toward osteoclast activation in the vicinity of BMLs, contributing to trabecular loss, sensitization, and the biomechanical changes that precede SBP failure [11,20,21].

This molecular dialogue also has clinical implications: markers of bone turnover such as C-telopeptide of type I collagen (CTX-I) and N-terminal propeptide of type I procollagen (PINP) and cartilage turnover markers such as urinary C-telopeptide of type II collagen (uCTX-II) and cartilage oligomeric matrix protein (COMP) integrate signals from both tissues and have been explored as prognostic biomarkers of OA progression [11,20]. The dependence of cartilage health on subchondral signaling explains why treatments limited to a single tissue compartment may be insufficient, and why interventions that modulate the shared microenvironment—rather than only its cartilage or bone components in isolation—are conceptually attractive.

### 2.3. Subchondral Bone and OA Pain

Chondrocytes are aneural, and clinical pain in knee OA correlates poorly with radiographic cartilage loss. Subchondral bone, by contrast, is richly innervated. Aso and colleagues have shown in symptomatic knee OA specimens that subchondral pathology—particularly increased NGF expression in osteochondral channels and elevated osteoclast density—is independently associated with symptomatic disease after adjustment for chondropathy and synovitis [7,8]. Subsequent work by the same group linked subchondral cyst-like lesions to increased osteoclastogenesis and sensory nerve growth, and reinforced the role of localization of subchondral marrow lesions in weight-bearing pain [8,9]. Immunohistochemical characterization has repeatedly identified nerve fibers growing within newly formed vascular channels, with local expression of NGF and its receptor tropomyosin receptor kinase A (TrkA) on osteoclasts and osteoblasts—an anatomical substrate through which subchondral remodeling can be directly perceived as joint pain [7,8,9,20]. Sensory nerve fibers in these channels co-express calcitonin gene-related peptide and substance P, consistent with a nociceptive phenotype capable of contributing to peripheral sensitization in painful OA [7,8,9,20].

Reviews of the subchondral bone microenvironment place these findings within a broader model in which abnormal remodeling, angiogenesis, and sensory innervation together drive OA pain and structural progression [20]. Anti-NGF therapies, although constrained by rare joint-safety signals, have independently supported a causal role for NGF signaling in OA pain and reinforce the therapeutic logic of modulating the subchondral compartment [20,25]. Complementary evidence from bisphosphonate trials and observational data suggests that modulation of subchondral osteoclastic activity can influence pain trajectories, particularly in patients with prominent BMLs, although structural benefits have been more variable [11,20]. Interventions that converge on the subchondral compartment—whether pharmacological, biological, or mechanotransductive—may therefore target a shared nociceptive and structural substrate rather than isolated pathways.

Taken together, the osteochondral unit provides both a mechanical rationale (load transfer and interface integrity) and a biological rationale (cross-talk, remodeling, and pain generation) for treating the subchondral bone directly rather than solely acting on cartilage. From a bioengineering perspective, this compartment simultaneously determines the boundary conditions for cartilage repair and constitutes an accessible target for combined mechanical and biological intervention.

## 3. Bone Marrow Lesions: More than an MRI Signal

BMLs represent the most widely recognized imaging biomarker of subchondral bone involvement in knee OA. On fluid-sensitive MRI sequences—typically fat-suppressed proton density, T2, or short-tau inversion recovery—BMLs appear as ill-defined regions of increased signal within the marrow compartment [4,26]. The original term “bone marrow edema” is now widely regarded as a misnomer, because histological correlation demonstrates that free fluid accounts for only a small fraction of the signal abnormality. Histologically, BMLs correspond to a heterogeneous mixture of fibrosis, necrosis, remodeling trabeculae, microdamage, and, in more severe stages, microfractures [4,26]. High-signal regions frequently contain immature woven bone, increased osteoid volume, marrow fibrosis, and infiltrates of inflammatory cells and vascular tissue [7,8,9,20]. Increased osteoclast activity, elevated NGF expression, and sprouting sensory nerves within osteochondral channels connect these subchondral changes directly to the mechanisms of OA pain and progression [7,8,9,20]. Semiquantitative scoring systems such as the MRI Osteoarthritis Knee Score (MOAKS) have standardized the reporting of BML size, number, and location, enabling more consistent comparison across studies and clinical cohorts [26]. Quantitative volumetric assessment of BMLs, whether manual or automated, has further improved sensitivity to longitudinal change, and now serves as an outcome measure in several clinical trials of bone-active and biological therapies in knee OA [4,5,26].

A further clinically important observation is that BMLs are not a homogeneous entity along the axis of reversibility, and that the subchondral compartment can be conceptualized as evolving through four sequential stages that anticipate the schema presented in Section 4.4, with working imaging definitions summarized in Table 1. In Stage 1, the lesion is biologically active but the SBP remains structurally preserved, the articular surface is intact, and the overlying articular cartilage is typically of full thickness; such lesions are, in principle, potentially reversible, and serial MRI studies have documented spontaneous or treatment-associated regression when mechanical overload is removed or when the underlying stimulus is modified [4,5]. In Stage 2, the SBP remains continuous at the resolution of routine imaging but sustains microstructural compromise—microdamage, increased intracortical porosity, and early ingress of fluid through microscopic channels or microcracks that are below the resolution of routine MRI—and the overlying articular cartilage may begin to thin. Histological studies of BMLs have documented exactly such changes—trabecular microfracture, marrow fibrosis, and sclerotic but less well mineralized bone—beneath an imaging-continuous plate [4,26,27,28,29], which is why this phase is frequently under-recognized on routine MRI and plain radiographs. It may nonetheless be suggested indirectly, for example by focal, well-circumscribed fluid-signal foci or incipient micro-cystic change within an ill-defined active BML on fluid-sensitive sequences, that are smaller than the cyst-like lesions defining Stage 3 and show no visible communication with the joint space; lesions in this latent phase are already at meaningfully elevated risk of progression toward irreversible structural failure [30,31]. We emphasize that Stage 2 is a hypothesized latent phase inferred from histological evidence [4,26,27,28,29] rather than an objectively diagnosable imaging entity; its prospective identification will require dedicated validation with high-resolution imaging protocols. In Stage 3, discontinuity of the SBP—interruption of the plate evident on routine MRI or computed tomography (CT), typically accompanied by subchondral cyst-like lesions—becomes the defining feature, and the overlying articular cartilage is often substantially thinned or focally lost, while the underlying bony articular contour is still preserved and has not yet undergone frank collapse; from this stage onward, continued unprotected loading tends to culminate in articular surface collapse, and the capacity for biological reversal is markedly reduced [30,31,32]. In this schema, the boundary between Stage 2 and Stage 3 is defined by SBP continuity: in Stage 2 the plate is compromised but continuous, whereas in Stage 3 it is interrupted. Stage 4 represents overt articular surface collapse, usually with extensive loss of the overlying articular cartilage: the fully irreversible endpoint at which macroscopic joint geometry can no longer be restored by biological or mechanotransductive means and surgical reconstruction becomes the dominant therapeutic option.

From a treatment standpoint, these observations imply that the biological and mechanical windows for meaningful joint preservation narrow substantially between Stage 2 and Stage 3, and that recognizing Stage 2—a phase that is frequently under-recognized—before SBP discontinuity and cyst formation emerge is central to any subchondral-directed treatment strategy. In the present review, this four-stage reversibility continuum is used as a conceptual scaffold rather than as a formal classification, and is intended to capture the progressive loss of the overlying articular cartilage alongside the deterioration of the SBP and articular surface. Cartilage thickness and subchondral stage are correlated but not interchangeable: the stages are defined by SBP integrity and articular contour, not by cartilage thickness, which is why a KL grade 4 knee may occupy any pre-collapse stage—in practice most often Stage 2 and 3 (Section 6.3). The overarching clinical implication is unambiguous: because Stage 1 is potentially reversible and Stage 2 already carries a substantially elevated risk of irreversible progression, subchondral-directed treatment is hypothesized to achieve its greatest joint-preserving benefit when it is delivered before SBP discontinuity and cyst formation become imaging-overt and the lesion crosses into the irreversible phase, and this early treatment imperative is one of the two central messages that this review carries through the subsequent sections.

Clinically, BMLs are strongly associated with pain: approximately 80% of patients with painful knee OA exhibit BMLs, whereas only about 30% of asymptomatic individuals do [6]. Systematic reviews confirm that people with knee OA who have BMLs are two- to five-fold more likely to have knee pain than those without, and that the presence, size, and progression of BMLs predict incident radiographic OA and future joint replacement [4,5]. Recent longitudinal analyses from large cohorts have shown that BML progression is associated with a substantially higher risk of developing radiographic OA (hazard ratio approximately 3.0 in one large analysis) [33]. BMLs also frequently coexist with subchondral bone attrition and meniscal extrusion, further raising the risk of progression [12]. Their location—particularly in weight-bearing femoral and tibial regions—correlates with pain topography, and shifts in BML location have been associated with shifts in the site of maximum pain, indicating a dynamic, clinically meaningful biological process [4,5,12]. In advanced disease, coalescence of BMLs, subchondral cyst formation, and cortical irregularities on the articular surface frequently coexist and jointly predict short-term structural deterioration [12,19,26].

Importantly, BML activity is dynamic. Serial imaging demonstrates that BML volume can change substantially over weeks to months in the same individual, indicating a modifiable biological process [34]. This reversibility—together with the histological evidence of active remodeling rather than passive edema—provides a mechanistic rationale for interventions that address angiogenesis, remodeling balance, and cellular signaling simultaneously. Studies of exercise, weight loss, bisphosphonates, and, more recently, ESWT and intra-osseous orthobiologics have reported measurable reductions in BML volume, with variable degrees of accompanying symptomatic benefit [11,20,35,36,37,38,39]. Understanding BMLs as composite biological lesions—rather than as marrow edema in a mechanical sense—reframes them as candidate targets for combined mechanotransductive and biological therapy rather than as fixed radiological findings.

## 4. The Subchondral Bone Plate as a Structural Turning Point

### 4.1. Anatomy and Biomechanics of the SBP

The SBP is the thin cortical bone lamella immediately beneath the calcified cartilage. It anchors the calcified interface and transfers stress into the trabecular network. Its integrity is therefore a prerequisite for physiological load transfer across the osteochondral unit [13,14]. In OA, the SBP undergoes complex, region-dependent remodeling: it may thicken and become less porous in certain regions while thinning, becoming more porous, or focally interrupted in others [3,11]. Regional heterogeneity is particularly marked at the medial tibial plateau in varus knees and at the medial femoral condyle in cases of subchondral insufficiency fracture, mirroring the local load distribution and clinical topography of pain [12,19,26]. Micro-CT and histomorphometric studies have documented that OA-affected SBP shows increased mean thickness with paradoxically elevated intracortical porosity, altered mineralization, and marked cell-scale changes—including increased osteocyte lacunar density and disorganized collagen orientation—that jointly reduce the mechanical quality of the plate even when its bulk dimensions increase [3,11,20]. In the language of hard-tissue bioengineering, the SBP is the load-bearing plate of the osteochondral construct; changes in its geometry and material properties propagate mechanically into both the overlying cartilage and the underlying trabecular network, and are difficult to counteract with cartilage-only interventions [15,16,17]. This has practical implications for regenerative strategies: if the plate is mechanically compromised, even a well-formed cartilage neotissue is exposed to abnormal stress concentration and may not achieve durable integration.

### 4.2. From Structural Deterioration to Disruption

Beyond diffuse changes in thickness and porosity, focal cortical disruption of the SBP is increasingly recognized. MRI-based descriptions of subchondral insufficiency phenomena—such as stress-related low-signal lines within a BML, subchondral fluid clefts, and localized cortical irregularities—reflect underlying failure of the plate under repetitive load [4,19,26]. Subchondral insufficiency fractures of the knee (SIFK), in particular, are characterized by a focal low-signal line adjacent to the articular surface within a BML and are associated with a substantial risk of subsequent articular surface collapse and rapid clinical deterioration [19]. Risk factors for progression include older age, low bone mineral density, medial meniscal root tears with meniscal extrusion, obesity, and persistent malalignment, each of which alters local mechanics or bone quality in ways that favor plate failure [11,12,19,26]. Once such focal disruption develops without overt articular surface collapse, the joint enters a distinct structural state: mechanical integrity is compromised, but the bony articular contour is still preserved and the cartilage envelope is variably affected. Biologically, this state may still allow effective remodeling if the local vascular and cellular environment is favorable, providing a rationale for intervening before irreversible geometric change. From a bioengineering perspective, this state resembles a scaffold with a focal defect that has not yet lost its overall geometric integrity—an intermediate condition in which biological and mechanical inputs may still be capable of restoring function. Subchondral insufficiency fracture and localized osteonecrosis represent related but distinct pathways that can enter this continuum: at Stage 2 while the plate remains continuous on imaging (a subchondral fracture line beneath an uninterrupted SBP), and at Stage 3 once interruption of the plate becomes apparent. In both entities the overlying cartilage is frequently intact at presentation, yet the risk of subsequent articular surface collapse is high [19,32]. They are therefore considered within the same pre-collapse therapeutic logic, although the present staging is formulated for OA-related BMLs.

### 4.3. Articular Surface Collapse and Its Consequences

When SBP discontinuity is severe or persistent under continued loading, the overlying articular surface may collapse. Once collapse occurs, the mechanical support upon which cartilage depends is lost, joint congruity is altered, and secondary changes accelerate throughout the joint [3,11]. The mechanical trajectory after articular collapse is difficult to reverse with biological or mechanotransductive interventions alone, because restoration of macro-scale articular geometry falls outside the effect range of current orthobiologics and of external shock wave stimulation. In advanced cases, collapse is often associated with progressive limb malalignment, meniscal insufficiency, and secondary ligamentous laxity, further increasing local stress and accelerating cartilage loss [3,11,19]. Our recent retrospective clinical study of patients with knee OA and BMLs treated with ESWT alone, APS + ESWT, or MSC-based therapy + ESWT (intra-articular alone or combined intra-articular/intra-osseous) provides observational support for this concept [18]. Across four treatment strategies, the pattern of clinical response consistently tracked subchondral structural status rather than the type of biological product used: patients without articular surface collapse showed markedly greater Knee Injury and Osteoarthritis Outcome Score (KOOS) improvements across arms, whereas patients with articular surface collapse showed minimal or negligible KOOS gains even when biological and mechanical therapies were combined (Table 2) [18]. This observation is consistent with the mechanical reasoning above and reinforces the importance of intervening before articular geometric failure occurs. Importantly, this final collapse is typically not an isolated event but the terminal step of a progressive cascade in which coalescing BMLs, loss of SBP continuity, and incident subchondral cyst-like lesions precede macroscopic articular incongruity [30,31]. Once SBP discontinuity or cyst coalescence is established, the lesion has moved substantially toward the irreversible end of the disease spectrum, and the structural benefit achievable with biological or mechanotransductive interventions diminishes accordingly [31]. Interrupting this cascade before SBP discontinuity becomes evident therefore represents the single most consequential opportunity for joint preservation.

### 4.4. A Potential Pre-Collapse Therapeutic Window

**Figure 2:**
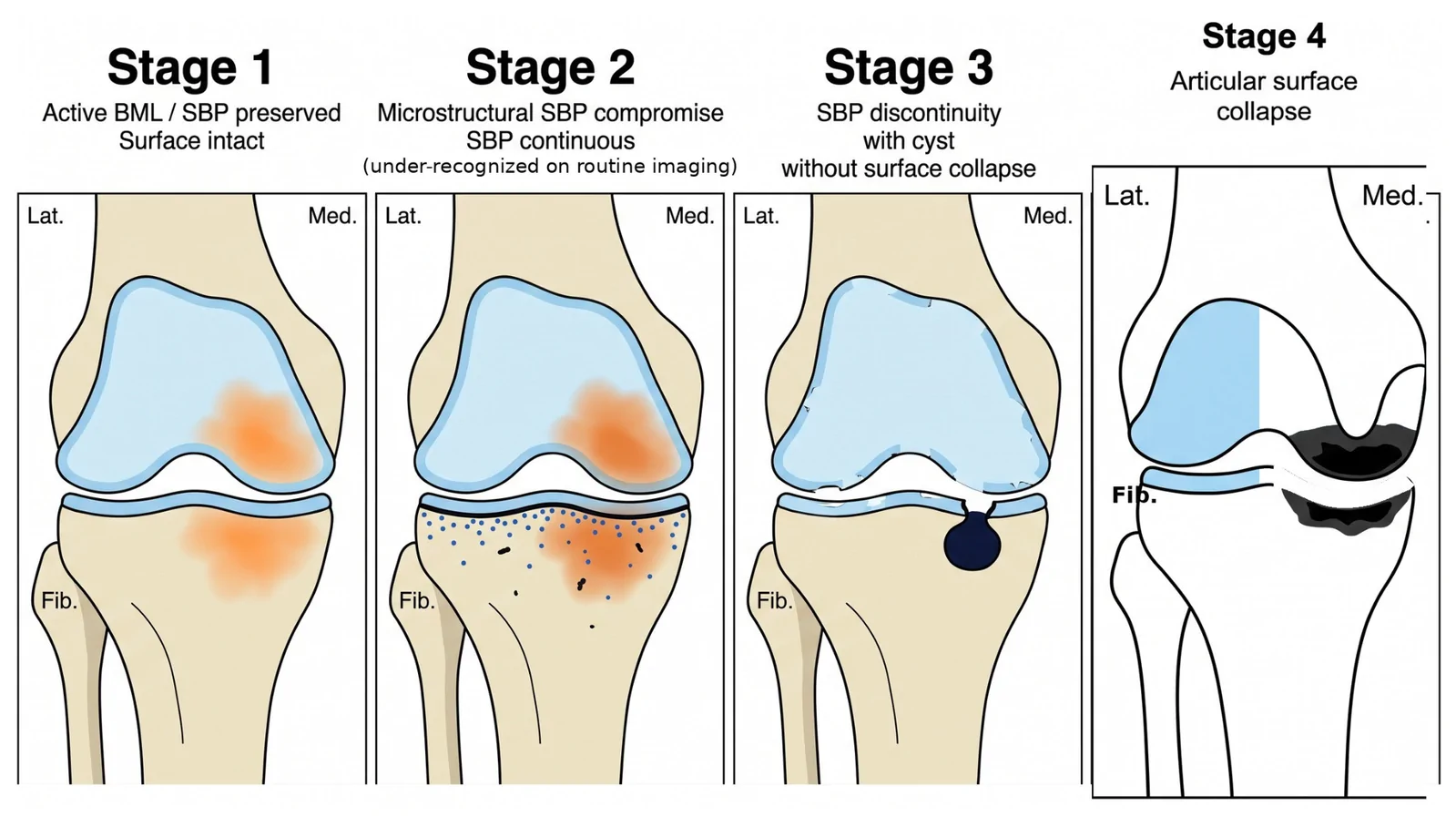
Conceptual progression of subchondral pathology from an imaging perspective. Four schematic stages illustrate a pathophysiologically ordered continuum. Stage 1: Biologically active bone marrow lesion with the subchondral bone plate (SBP) still structurally preserved, the articular surface intact, and the overlying articular cartilage typically of full thickness—a phase in which the lesion is, in principle, potentially reversible. Stage 2: Microstructural compromise of the SBP with the plate still continuous on imaging, fluid ingress through microscopic channels below imaging resolution, and possible early thinning of the overlying articular cartilage; because the plate remains continuous, this phase is frequently under-recognized on routine MRI and plain radiographs, yet it already carries a meaningfully elevated risk of irreversible progression, and is presented here as a hypothesized latent phase rather than a diagnosable imaging entity. Stage 3: Discontinuity of the SBP evident on routine MRI or computed tomography (CT), typically accompanied by subchondral cyst-like lesions, with the overlying articular cartilage often substantially thinned but with the bony articular contour still preserved (no frank surface collapse yet); from this stage onward the achievable regenerative benefit is substantially reduced. Stage 4: Overt articular surface collapse, usually with extensive loss of the overlying cartilage, representing the terminal, fully irreversible endpoint that typically warrants surgical reconstruction. Stages 1–3 jointly define the pre-collapse phase that is proposed as the therapeutic window in the present review, whereas the post-collapse phase (Stage 4) is associated with limited regenerative benefit. The stages shown are descriptive only and do not constitute a formal or validated grading system. The four imaging-based stages shown here map onto the three-stage therapeutic concept in Section 8 as follows: Stage 1 corresponds to Stage A, Stage 2 to the core of Stage B (the principal target of the pre-collapse therapeutic window), Stage 3 to late Stage B with diminishing benefit, and Stage 4 to Stage C. Abbreviations: BML, bone marrow lesion; SBP, subchondral bone plate; MRI, magnetic resonance imaging; CT, computed tomography. Fib., fibula; Lat., lateral; Med., medial. The orange shading represents BMLs; the blue dots and black marks in Stage 2 schematically represent microscopic fluid ingress/intracortical porosity and microdamage or microcracks, respectively; the dark blue area in Stage 3 represents a subchondral cyst-like lesion; and the black area in Stage 4 represents articular surface collapse.

The natural history of subchondral pathology may be summarized as a continuum: Stage 1—biologically active BML with preserved SBP and typically full-thickness overlying cartilage (potentially reversible) → Stage 2—microstructural SBP compromise with the plate still continuous on imaging, microscopic fluid ingress beneath an imaging-continuous plate, and possible early thinning of the overlying cartilage, frequently under-recognized on routine imaging but already at elevated risk of irreversible progression → Stage 3—imaging-evident SBP discontinuity, typically with subchondral cyst formation, with the overlying cartilage often substantially thinned yet with the bony articular contour still preserved → Stage 4—articular surface collapse, usually with extensive loss of the overlying cartilage, representing the fully irreversible endpoint. Each transition may progressively narrow the achievable benefit of biological interventions. The entire pre-collapse phase may therefore constitute a therapeutic window for combined mechanical and biological interventions, and Stage 2 in particular—microstructural SBP compromise that has not yet progressed to SBP discontinuity—is likely the point within this window at which such interventions have their greatest incremental opportunity to preserve joint function (Figure 2). Importantly, this framing treats stage transitions as biologically and mechanically continuous rather than dichotomous, and does not depend on any single grading system; it can be operationalized using standardized MRI scoring of BML activity, SBP contour, and articular surface integrity together with clinical measures. Framed in bioengineering terms, the window corresponds to a state in which the load-bearing scaffold of the joint retains sufficient geometric integrity for biological and mechanotransductive inputs to yield structural benefit, rather than palliation alone. This concept requires prospective validation but provides a testable framework for future stratified trials.

The relationship of this continuum to established instruments deserves explicit comment. MOAKS [26] is a validated semi-quantitative system for scoring individual MRI features (including BML size and number, cartilage morphology, and cysts); the present four-stage schema is not an alternative scoring system but a conceptual ordering of subchondral structural integrity that can be described using MOAKS-type feature scores (BML activity, SBP contour, cyst-like lesions, and articular surface integrity) together with thin-slice CT where available (Table 1). Subchondral insufficiency fracture and localized osteonecrosis are distinct diagnostic entities that can enter the continuum at Stage 2 or Stage 3 depending on plate continuity (Section 4.2) [19,32], and subchondral cyst-like lesions are the characteristic imaging accompaniment of Stage 3 [30,31]. The working imaging definitions of each stage and of the stage transitions are summarized in Table 1.

## 5. ESWT as Mechanical/Biophysical Stimulation of Subchondral Bone

### 5.1. Beyond Analgesia: A Mechanotransductive Intervention

Focused shock waves deliver high-amplitude, short-duration pressure pulses to targeted tissues, generating acoustic and cavitational stresses that are transduced into biological signals by cells in the treated field. Cheng and Wang described the biological mechanism of shock wave in bone as fundamentally mechanotransductive, involving membrane deformation, cytoskeletal remodeling, and downstream molecular signaling through integrin/focal adhesion kinase and mitogen-activated protein kinase (MAPK) pathways [23]. Focused ESWT differs from radial pressure wave systems in that its energy is delivered to a defined focal zone at controllable depth, which is particularly relevant when targeting deep structures such as the subchondral compartment beneath a diseased knee [23,24]. Radial pressure waves, by contrast, deliver energy over a broader superficial field and are generally less suitable for deep bone targeting. Energy flux density, total pulse number, and session frequency together determine the biological dose; parameter selection for the subchondral compartment differs from that for soft-tissue applications and is a focus of ongoing standardization efforts [23,24,35,36]. Representative treatment parameters from clinical studies, together with our own protocol, are summarized in Table 3 (see Section 5.3). ESWT can therefore be viewed as a non-invasive mechanical actuator that engages the very mechanotransduction pathways central to hard-tissue engineering—the same pathways that scaffold designers try to recruit through matrix stiffness, topography, and dynamic loading [15,23,24].

### 5.2. Angiogenesis and Microcirculation

Preclinical and clinical studies have consistently shown that ESWT upregulates VEGF and endothelial nitric oxide synthase (eNOS), promoting angiogenesis and improved microcirculation in poorly vascularized tissues [23,24]. This vascular response is particularly relevant to the subchondral compartment, where perfusion is normally limited and where reduced blood flow has been implicated in BML pathophysiology and in avascular conditions such as osteonecrosis [20,23,35]. Contrast-enhanced imaging and laser Doppler studies in animal models have shown increased regional perfusion within hours to days after focused ESWT, with sustained neovascularization documented histologically over weeks [24]. In parallel, ESWT increases the expression of proliferating cell nuclear antigen and stromal cell-derived factor 1 (SDF-1)/C-X-C chemokine receptor type 4 (CXCR4) signaling in treated bone, consistent with mobilization of progenitor cells to the treated field [24,40,41]. Because vascularization is a well-recognized rate-limiting factor in bone regeneration and in engineered hard-tissue constructs, ESWT-induced angiogenesis represents a mechanistic bridge between mechanical stimulation and effective delivery of biological signals [15,16,23,24]. In the setting of BMLs, in which local perfusion is already abnormal, this pro-angiogenic action provides a plausible mechanism for observed reductions in BML volume after ESWT [35,36,37].

### 5.3. Osteogenic and Remodeling Effects

Experimental data indicate that ESWT stimulates osteoblast differentiation, enhances mineralization, and promotes bone formation through mechanotransductive pathways including BMP-2, Wnt/β-catenin, and Runx2-mediated signaling [24]. In parallel, moderate-energy ESWT modulates osteoclast activity and shifts the RANKL/OPG balance toward more coordinated remodeling, which may be particularly relevant in the context of BMLs and SBP deterioration where remodeling is uncoupled [24]. Histological analyses of treated bone have shown increased trabecular thickness, improved trabecular connectivity, and reduced empty osteocyte lacunae after ESWT in preclinical models of osteonecrosis and delayed fracture healing, consistent with a shift from resorption-dominated to formation-dominated remodeling [24,42,43]. Clinically, ESWT has demonstrated the ability to reduce BML volume and pain in conditions such as primary bone marrow edema syndrome of the knee and osteonecrosis of the femoral head, with several studies reporting significant improvements in pain and functional scores and radiological evidence of BML resolution over months [35,36,37,42,43,44]. In OA-related BMLs, clinical data are currently limited largely to symptomatic outcomes, and lesion volume reduction remains to be confirmed in adequately designed human studies; trial heterogeneity in energy flux density, pulse number, and anatomic targeting limits direct comparison [35,36,37]. Clinical studies of ESWT in knee OA generally report short- to mid-term improvements in pain and function with a favorable safety profile, while emphasizing the need for standardized protocols and longer follow-up [35,36,37,38].

**Table 3:** ESWT parameters in representative studies of knee OA and related subchondral conditions.

Study	Condition (Design)	Modality/Device	EFD (mJ/mm^2^)	Pulses × Sessions (Interval)	Freq. (Hz)	Targeting
Nakasato et al., 2025 [18]	Knee OA with BML (retrospective observational)	Focused, electromagnetic (DUOLITH SD1, STORZ MEDICAL)	0.10–0.25, titrated to tolerance	2500 (3500 in APS/MSC-combination sessions) × ≥3 at 2-week intervals, then every 2–4 weeks until clinical response (mean 4.4 ± 1.3 by 3 M; 6.0 ± 2.4 by 6 M)	4	BML localized on MRI; target set under ultrasound with knee flexed (±fluoroscopy); three-dimensional lesion coverage
Zhao et al., 2013 [36]	Knee OA (RCT vs. sham, *n* = 70)	Focused	0.25	4000 × 4 (weekly)	NR	Regional application to affected knee
Gao et al., 2015 [44]	Primary bone marrow edema syndrome of the knee (RCT, *n* = 40)	Focused, electromagnetic (Dornier Compact DELTA II)	>0.44 (high energy)	3000–4000 × 2 (1-week interval)	2–3	Radiographic guidance to lesion region
Vulpiani et al., 2012 [42]	Early femoral head osteonecrosis (prospective, long-term)	Focused	0.50	2400 × 4 (48–72-h intervals)	NR	Imaging-guided to necrotic zone
Avendaño-Coy et al., 2020 [37]	Knee OA (meta-analysis of RCTs)	Focused and radial across trials	0.05 (low) to >0.25 (high); most trials 0.08–0.25	1000–2500 per session (4000 in [36]); typically weekly, 4–8 sessions	4–12	Landmark-based or point of maximum pain
Wang et al., 2017 [35]—preclinical (rat)	Early knee OA model (ACLT + meniscectomy)	Focused	0.25	800 × 1 per site	—	Medial femoral and tibial condyle subchondral bone (medial tibia most effective)

EFD, energy flux density; NR, not reported; RCT, randomized controlled trial; ACLT, anterior cruciate ligament transection. The Wang et al. row is preclinical and is included because it informs anatomical targeting of the subchondral compartment; all other rows are clinical. Heterogeneity in modality, energy, pulse number, session schedule, and targeting is a plausible source of inter-study variability and underscores the need for standardization (Section 5.1 and Section 9).

### 5.4. Modulation of Inflammation and Mesenchymal Cell Activity

ESWT modulates local inflammatory signaling and increases the recruitment, proliferation, and osteogenic differentiation of mesenchymal progenitors [23,24,45]. Anti-inflammatory effects include downregulation of pro-inflammatory cytokines (interleukin-1β (IL-1β), tumor necrosis factor-α (TNF-α), and interleukin-6 (IL-6)) and shifting of macrophage polarization toward reparative M2 phenotypes in preclinical models [24,46]. Concurrently, ESWT enhances migration of bone marrow- and adipose-derived MSCs toward treated regions and primes their osteogenic and angiogenic secretome, with increases in the expression of osteogenic transcription factors and angiogenic growth factors [41,47]. In vitro, moderate-energy ESWT on MSC cultures has been shown to enhance proliferation and to bias differentiation toward osteogenic and chondrogenic lineages without compromising viability, consistent with the notion that shock waves act as a mechanotransductive priming stimulus [24,45,48]. The same mechanotransductive pathways that promote bone healing may therefore create a microenvironment more receptive to exogenously delivered biological products—a concept that has direct implications for combination therapy design and echoes the ‘signals + cells + mechanical cues’ triad central to hard-tissue engineering [15,16,17].

### 5.5. Summary

Mechanical/biophysical stimulation by ESWT can be plausibly framed as a means of conditioning the subchondral compartment—improving vascularity, mobilizing mesenchymal responses, and shifting remodeling balance—rather than merely providing analgesia. Angiogenic, osteogenic, and immunomodulatory effects are supported directly by experimental data, whereas some downstream implications for combined regenerative therapy remain hypothetical and require dedicated clinical evaluation. Positioned in this way, ESWT is not simply a symptomatic adjunct but a biologically active preparatory intervention that may enhance the response to orthobiologic therapy.

## 6. Regenerative Medicine for the Osteochondral Unit

### 6.1. PRP and APS

PRP is an autologous concentration of platelets suspended in plasma and enriched in growth factors (platelet-derived growth factor (PDGF), TGF-β, insulin-like growth factor 1 (IGF-1), VEGF, epidermal growth factor (EGF), and fibroblast growth factor (FGF)). Multiple randomized trials and meta-analyses in knee OA have shown intra-articular PRP to be superior to hyaluronic acid or placebo for pain and function over 6 to 12 months, with a favorable safety profile [49,50]. Product characteristics matter: leukocyte-poor PRP formulations have been associated with more consistent symptomatic benefit than leukocyte-rich formulations, and platelet dose, activation, and preparation kit are all sources of clinical heterogeneity [49,50,51]. Systematic reviews have additionally suggested that lower joint radiographic severity (e.g., KL grade 2 and 3) and younger patient age may be associated with greater symptomatic response to PRP, although these observations require prospective confirmation [49,50]. Standardization of PRP reporting according to platelet count, leukocyte composition, concentration factor, and activation status is now recommended to improve comparability across trials [51].

APS is a related autologous product enriched in anti-inflammatory cytokines (including IL-1 receptor antagonist, soluble TNF receptors, and soluble IL-1 receptor II) as well as growth factors [52]. Randomized and observational studies of APS have reported symptomatic benefit in knee OA at follow-up intervals of up to three years, with a safety profile comparable to that of intra-articular PRP [52]. In our own clinical experience, APS combined with ESWT contributes to accelerated symptom improvement in patients with prominent synovitis [18]. Importantly, both PRP and APS act primarily through the intra-articular compartment—synovium, cartilage surface, and joint fluid—and their access to deeper subchondral compartments is limited by the SBP itself. This anatomical limitation is a recurring theme in the rationale for intra-osseous delivery.

### 6.2. MSCs

MSCs from bone marrow, adipose tissue, or synovial tissue exert their effects predominantly through paracrine signaling—including secretion of anti-inflammatory cytokines, growth factors, and extracellular vesicles—rather than through direct engraftment [53,54,55]. Meta-analyses of randomized trials have reported significant improvements in pain and function compared with control interventions, with acceptable short-term safety profiles [53,54,55]. Product characteristics that plausibly influence outcome include tissue source (bone marrow, adipose, umbilical, synovial), culture expansion versus point-of-care preparations, cell dose per injection, and immunophenotypic and functional characterization [53,54,55,56]. Bone marrow-derived MSCs show robust osteogenic and chondrogenic potential and are the most extensively studied source in the knee OA literature, whereas adipose-derived MSCs offer greater ease of harvest and higher yield [53,54,55]. Synovial MSCs have shown particular affinity for cartilage in preclinical work, and umbilical-derived MSCs offer allogeneic scalability, although with additional regulatory and immunological considerations [53,54,55,56]. Evidence quality across trials is heterogeneous, and standardization of cell source, dose, delivery vehicle, and outcome measures remains an active area of investigation [55,56]. Culture-expanded products offer more consistent cell numbers and quality control but face regulatory complexity, whereas point-of-care preparations such as bone marrow aspirate concentrate (BMAC) provide operational simplicity at the cost of variable cell content [39,55,56,57,58]. MSCs are therefore best viewed not as replacement cells but as biological modulators whose secretome shapes local remodeling and immune tone. The principal orthobiologic product classes, their composition, the clinical evidence supporting each, and their proposed stage assignment within the framework of Section 8 are compared in Table 4.

### 6.3. Intra-Articular Versus Intra-Osseous Delivery

Sánchez, Delgado, and colleagues introduced the concept of combining intra-articular with intra-osseous PRP infiltration to target both the synovial–cartilage compartment and the subchondral compartment in severe knee OA [59]. In their procedural description, PRP is delivered under fluoroscopic or ultrasound guidance into the medial femoral condyle and medial tibial plateau after a standard intra-articular injection, using a bone trocar placed within the subchondral zone [59]. Clinical series and cohort studies have suggested improved 6- and 12-month outcomes with the combined approach compared with intra-articular PRP alone in advanced disease [60,61]. A translational study by Ganguly and colleagues further demonstrated that combined intra-osseous and intra-articular PRP infiltration alters the frequency and behavior of subchondral bone MSCs in patients with hip OA, providing mechanistic support that intra-osseous delivery reaches biologically relevant progenitor populations [62]. Most recently, a double-blind, multicenter, randomized trial in 86 patients with KL grade 3–4 knee OA compared intra-osseous plasma rich in growth factors (PRGF) with intra-osseous saline, both followed by three intra-articular PRGF injections; the intra-osseous PRGF group showed significantly greater improvement in nearly all KOOS and Western Ontario and McMaster Universities Osteoarthritis Index (WOMAC) domains at 3, 6, and 12 months, with no serious adverse events [63]. This placebo-controlled design isolates the contribution of the subchondral route itself and substantially strengthens the rationale for intra-osseous delivery in advanced disease.

In addition, intra-osseous MSC delivery has recently attracted attention as a strategy for placing MSCs directly within subchondral BMLs and peri-SBP regions, so that their secretome can act within the subchondral microenvironment rather than only within the joint cavity [39,53,54,55,64]. In our own retrospective clinical observations, the treatment arm that added intra-osseous MSC administration to intra-articular MSC + ESWT (MSC-B) showed the most favorable outcome trend in patients with SBP disruption but without articular surface collapse [18]. (“SBP disruption” in the terminology of [18] denotes imaging-evident interruption of the plate, i.e., Stage 3 in Table 1) Analogous approaches with BMAC, delivered both intra-articularly and into subchondral BMLs, have been explored by Kon and colleagues with safety and encouraging short- to mid-term clinical and radiological outcomes reported [39], as summarized in recent reviews [57,58]. Longer-term evidence comes from a randomized study by Hernigou and colleagues in patients with bilateral knee OA, in which subchondral BMAC injection was compared with intra-articular injection in contralateral knees: at 2 years, MRI showed slower structural progression in the subchondral group, and at 15 years the proportion of knees converted to arthroplasty was markedly lower after subchondral delivery (12 of 60 knees (20%) vs. 42 of 60 knees (70%)) [64]. These results derive from a single-center, single-surgeon series with a bilateral within-patient design, and require independent replication.

**Table 4:** Orthobiologic products: composition, principal clinical evidence, and proposed stage assignment within the framework of Section 8.

Product	Source/Processing	Key Composition	Routes Studied in Knee OA	Principal Clinical Evidence	Proposed Stage (Rationale)
PRP (leukocyte-poor/leukocyte-rich)	Autologous peripheral blood; point-of-care centrifugation	Concentrated platelets; PDGF, TGF-β, IGF-1, VEGF, EGF, FGF; LP vs. LR differ in leukocyte content	Intra-articular (1–3 injections); intra-osseous (femoral condyle + tibial plateau) in advanced OA [59,60,61,63]	RCTs/meta-analyses vs. HA/placebo: symptomatic benefit 6–12 M [49,50]; LP-PRP more consistent [51]; intra-osseous PRGF RCT positive at 12 M [63]	Stage A intra-articularly (synovial–cartilage compartment is the treatable biology); Stage B intra-osseously (route reaches the subchondral lesion)
APS	Autologous blood; two-step point-of-care processing	Anti-inflammatory cytokines (IL-1Ra, sTNF-R, sIL-1RII) plus growth factors	Single intra-articular injection [52]	RCT/observational benefit up to 3 years [52]; accelerated response with synovitis in [18]	Stage A (anti-inflammatory profile matched to synovitis-dominant disease)
MSCs (culture-expanded; bone marrow/adipose/synovial/umbilical)	Tissue harvest + culture expansion under regulatory oversight	Defined, characterized cell dose; paracrine/immunomodulatory secretome and extracellular vesicles	Intra-articular; intra-articular + intra-osseous (MSC-B in [18])	Meta-analyses: pain/function improvement, acceptable short-term safety [53,54,55,56]; combined IA + IO MSC + ESWT most favorable trend in Stage 3, non-collapsed knees [18]	Stage B (paracrine, immunomodulatory, and osteogenesis-supportive actions matched to subchondral remodeling imbalance; intra-osseous route required to reach the sub-SBP target)
BMAC	Autologous bone marrow aspirate; point-of-care concentration (no expansion)	Heterogeneous: MSC fraction (low, variable), platelets, growth factors	Intra-articular + subchondral/intra-osseous [39,64]	Prospective multicenter pilot: safe, encouraging outcomes [39]; randomized bilateral-knee series: less structural progression and fewer arthroplasties at 15 y after subchondral vs. intra-articular delivery [64]	Stage B (operationally simple intra-osseous option; long-term signal from [64] requiring independent replication)

Product classes are heterogeneous; evidence generated with one preparation cannot be assumed to generalize to another (platelet dose and leukocyte content for PRP; cell source, dose, and expansion status for MSC-based products). Stage assignments are testable proposals based on biological rationale and observational data [18], not evidence-based recommendations; no head-to-head randomized comparison between products at any stage exists. BM, bone marrow; HA, hyaluronic acid; IA, intra-articular; IO, intra-osseous; IL-1Ra, interleukin-1 receptor antagonist; LP/LR, leukocyte-poor/-rich; PRGF, plasma rich in growth factors; sTNF-R, soluble tumor necrosis factor receptor; sIL-1RII, soluble interleukin-1 receptor type II.

Taken together, these observations support the second central message of this review: even in radiographically advanced disease, including knees with end-stage KL grade 4 changes, joint preservation is not necessarily out of reach. The clinically relevant intervention here is the combination of two modalities acting together—intra-osseous orthobiologics (intra-osseous PRP, MSCs, or BMAC) and ESWT-mediated mechanotransductive conditioning—rather than orthobiologic delivery alone; durable structural benefit of intra-osseous delivery in advanced disease has been reported in independent series [59,60,61,63,64], and in our own series the combination of intra-osseous MSC with ESWT showed the most favorable trend in Stage 3, non-collapsed knees [18]. These data indicate that advanced radiographic OA per se should not be equated with an unconditional indication for arthroplasty. When the subchondral compartment remains viable, joint preservation through the two-modality combination of intra-osseous orthobiologics and ESWT should be considered before surgical reconstruction is committed to. Radiographic grade and subchondral structural stage are distinct axes: a KL grade 4 knee may still lie in the pre-collapse phase of the continuum described in Section 4.4 (most often Stage 2 and 3), with a preserved articular surface, and it is this structural status—not the radiographic grade—that is expected to govern the response to subchondral-directed therapy. We emphasize that the external long-term evidence [59,60,61,63,64] was generated with intra-osseous delivery alone; the addition of ESWT is proposed on mechanistic grounds (Section 5 and Section 7), on our own short-term observations [18], and on whether the combination outperforms intra-osseous delivery alone is untested and constitutes the first question for prospective trials.

Three outcomes must be kept distinct in interpreting this proposition: symptomatic improvement, structural preservation, and deferral or avoidance of arthroplasty. Current evidence supports symptomatic improvement for several of the component therapies (Section 5 and Section 6); evidence for structural preservation is preliminary; and evidence for arthroplasty avoidance is limited to a single long-term randomized series of subchondral BMAC [64] that requires independent replication—and no such evidence exists for the ESWT + orthobiologic combination. We also note that contemporary clinical practice guidelines [65,66,67] do not currently recommend PRP, cell-based therapies, or ESWT as standard care for knee OA, reflecting the heterogeneity of the underlying evidence; the present proposal is accordingly a research hypothesis to be pursued in selected patients within appropriate clinical governance, not a recommendation to substitute for guideline-based management.

The core hypothesis is straightforward: intra-articular biologics may not adequately reach lesions that lie beneath the SBP, whereas intra-osseous delivery—whether in the form of intra-osseous PRP, intra-osseous MSC, or intra-osseous BMAC—can, in principle, place biological signals directly within a subchondral BML or a peri-SBP zone. Whether this translates to superior structural or symptomatic outcomes across broader populations requires a further, adequately powered, randomized study [39,55,56,57,58]. Early comparative signals from cohort studies are encouraging but insufficient to establish intra-osseous delivery as a standard of care. Safety considerations specific to intra-osseous delivery—procedural pain, infection, and the theoretical risk of iatrogenic subchondral fracture in osteopenic bone—and to focused ESWT over subchondral bone, including the theoretical concern that shock waves delivered over a discontinuous plate or a cyst-like lesion could accelerate rather than arrest structural failure, have so far been reported as acceptable in the available series [18,39,59,60,61,62,63,64], but should be monitored prospectively.

### 6.4. What Regenerative Medicine Cannot Do Alone

However refined the delivery, biological products act on the microenvironment they encounter. If subchondral perfusion is poor, if the mechanical loading pattern remains pathological, or if the SBP is progressively failing under continued stress, biological signals may be insufficient to reverse the trajectory of disease. Cells or growth factors delivered into a mechanically hostile subchondral environment risk being consumed by ongoing inflammation or diverted by aberrant remodeling, rather than being effectively integrated into repair. Beyond the local microenvironment, systemic factors such as obesity, metabolic syndrome, and persistent mechanical malalignment further shape the response to biological intervention and cannot be corrected by orthobiologics alone [1,2,11]. This limitation motivates the pairing of biological regeneration with mechanical and biophysical conditioning—an approach that echoes the fundamental hard-tissue engineering principle that scaffolds, cells, and mechanical cues must be co-designed [15,16,17]. It also reinforces the case for stage-appropriate patient selection: biological interventions are most likely to yield structural benefit when the target compartment retains sufficient mechanical and vascular integrity to support repair.

## 7. Why Combine ESWT and Regenerative Medicine?

### 7.1. Complementary Modes of Action

The mechanistic case for combining ESWT with PRP/APS/MSC therapy rests on the recognition that these modalities act along complementary axes. Regenerative products deliver cells and molecular signals; ESWT modifies the mechanical and vascular environment in which those signals must operate. ESWT can enhance local perfusion (VEGF, eNOS), mobilize and prime mesenchymal progenitors, and promote osteogenic signaling (BMP, Wnt/β-catenin), thereby preparing a receptive microenvironment for biological products [23,24,41,45]. PRP and APS deliver a growth factor and anti-inflammatory cytokine milieu that can act on synovium, cartilage, and, when placed intra-osseously, on the subchondral marrow [18,49,50,51,52,59,60,61,62,63]. MSCs provide additional paracrine and immunomodulatory signaling and, when delivered both intra-articularly and intra-osseously (including intra-osseous MSC delivery), may reach both compartments of the osteochondral unit [18,39,53,54,55,56,57,58]. Sequencing considerations further motivate combination: applying ESWT shortly before orthobiologic delivery may prime the microenvironment and enhance responsiveness, whereas applying ESWT during follow-up may help consolidate biological effects; the optimal temporal relationship remains to be established [23,24].

**Figure 3:**
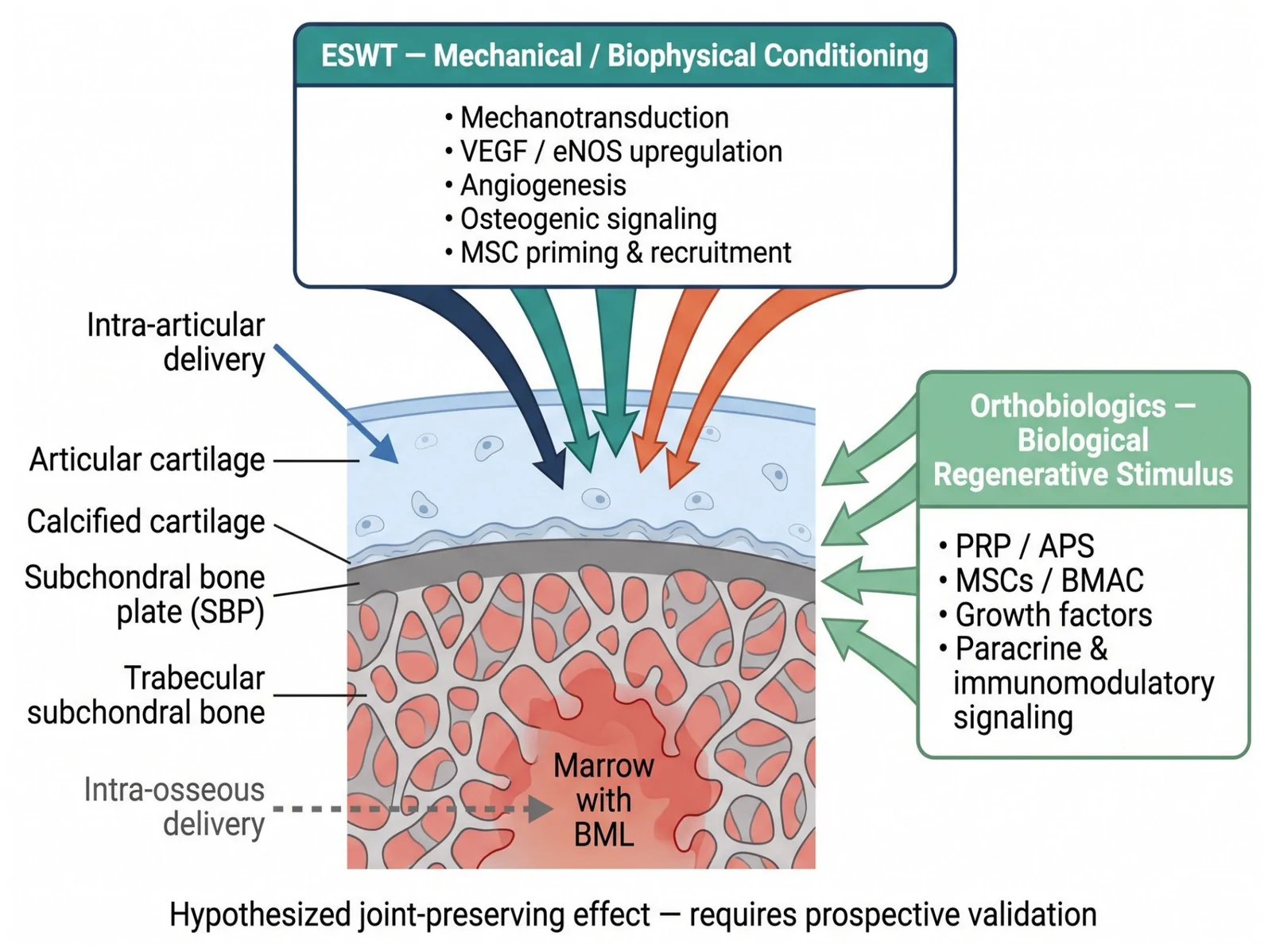
Combination therapy model for the osteochondral unit. Extracorporeal shock wave therapy (ESWT) provides mechanical/biophysical conditioning (mechanotransduction, VEGF/eNOS upregulation, angiogenesis, osteogenic signaling, and MSC priming and recruitment), while orthobiologics such as PRP and MSC-based products—with the same framework applying to APS and BMAC—provide a biological regenerative stimulus (growth factors and paracrine and immunomodulatory signaling). These two inputs converge, via intra-articular and, when appropriate, intra-osseous delivery, on a common target, the osteochondral unit, with a hypothesized joint-preserving effect that requires prospective validation. Abbreviations: ESWT, extracorporeal shock wave therapy; PRP, platelet-rich plasma; APS, autologous protein solution; MSC, mesenchymal stromal cell; BMAC, bone marrow aspirate concentrate; SBP, subchondral bone plate.

In bioengineering terms, ESWT and orthobiologics can be modeled as two inputs—one physical, one biochemical—converging on the same tissue construct, the osteochondral unit (Figure 3). This mirrors the co-design philosophy of contemporary hard-tissue engineering, in which scaffold architecture, biochemical cues, and mechanical stimulation are engineered together rather than in isolation [15,16,17]. Whereas engineered constructs rely on external bioreactors and implanted scaffolds to combine these inputs, ESWT combined with intra-articular and intra-osseous orthobiologics offers a minimally invasive analogue that operates directly on the native osteochondral unit. This positions the ESWT + orthobiologic combination as a translational bridge between conventional injection therapy and next-generation bioengineered constructs.

### 7.2. Existing Clinical Evidence

Direct high-quality randomized evidence for the combination of ESWT with PRP or MSC therapy in knee OA remains limited. Direct comparative studies of ESWT combined with intra-articular PRP in knee OA are scarce, and the available reports are small and heterogeneous in design [35,36,37,49,50]. In our recent retrospective clinical study of patients with knee OA and BMLs, four treatment strategies were compared: ESWT alone; APS + ESWT; intra-articular MSC + ESWT (MSC-A); and combined intra-articular and intra-osseous MSC + ESWT (MSC-B) [18]. Patients were stratified by subchondral structural status—specifically the presence or absence of SBP disruption and of articular surface collapse. Outcome was assessed with the KOOS at baseline and 3 and 6 months; adverse events were recorded prospectively [18]. The principal findings are summarized in Table 2.

ESWT alone yielded meaningful KOOS improvements in patients with BMLs but without SBP disruption or articular surface collapse. APS + ESWT tended to accelerate symptomatic improvement, with benefit particularly evident in patients with prominent synovitis. MSC-A + ESWT was associated with clinical improvement in patients whose pathology was dominated by intra-articular components. MSC-B + ESWT (with intra-osseous MSC delivery) showed the most favorable trend in patients with SBP disruption but without articular surface collapse; patients with articular surface collapse showed minimal improvement even with combined biological and mechanical therapy [18]. The authors explicitly framed these findings within the limits of a retrospective, non-randomized, single-center design, and cautioned against strong causal or comparative-effectiveness inferences [18]. Nevertheless, the pattern is consistent with the conceptual model presented here and provides an empirical starting point for prospective hypothesis testing. Convergent signals from independent intra-osseous PRP and BMAC series—particularly the observation of stage-dependent response and the biological plausibility of subchondral targeting—strengthen the case for formal randomized evaluation of stage-stratified combination therapy [39,57,58,59,60,61,62,63].

Several potential confounders further limit causal interpretation of the stage-dependent pattern in Table 2. The four arms differ in baseline KOOS, age, KL grade distribution, and sample size, and information on body mass index, mechanical alignment, BML volume, meniscal pathology (including root tears and extrusion), and synovitis burden was not uniformly available for adjustment. Most importantly, treatment allocation in [18] followed clinical characteristics and shared decision-making rather than randomization, so allocation bias could itself generate apparent stage-dependent differences: patients selected for the MSC-B strategy were, by design, those with the most advanced disease refractory to other options. Table 2 should therefore be read as hypothesis-generating pattern description, not comparative-effectiveness evidence, and the observed stage-dependence requires confirmation in prospective, stratified, and ideally randomized designs.

### 7.3. Strength-of-Evidence Framing

To avoid overstating what is currently known, we explicitly distinguish three tiers of evidence.

Tier 1—ESWT alone. Randomized trials and meta-analyses support short- to mid-term improvement in pain and function in knee OA [35,36,37,38], and randomized and prospective studies support BML/edema regression in bone marrow edema syndrome and early osteonecrosis [42,43,44]. Mechanistic effects—VEGF/eNOS upregulation, angiogenesis, osteoblast activation, BMP/Wnt signaling, and MSC priming—are directly supported by experimental data [23,24,40,41,45,46,47,48].

Tier 2—Orthobiologics alone. Randomized trials and meta-analyses support symptomatic benefit of intra-articular PRP [49,50,51], APS [52], and MSC-based products [53,54,55,56]; for the intra-osseous route, evidence includes cohort studies [59,60,61,62], one double-blind multicenter randomized trial of intra-osseous PRGF [63], and one long-term randomized series of subchondral BMAC [64].

Tier 3—The combination of ESWT with orthobiologic therapy. Direct evidence is currently limited to our retrospective, non-randomized, single-center observational series [18] in which the response pattern tracked subchondral structural status. No randomized trial of the combination has been conducted, and the proposition that the combination outperforms either modality alone is a hypothesis derived from Tiers 1 and 2 and mechanistic reasoning, not an established finding.

Author-proposed hypotheses requiring prospective validation. A pre-collapse therapeutic window in which combined mechanical and biological interventions maximize joint-preserving potential; and a stage-dependent selection of intra-articular versus combined intra-articular/intra-osseous delivery. The stage-dependent framework of Section 8 is therefore an extrapolation from single-modality evidence (Tiers 1–2) organized around the biology of the osteochondral unit; its purpose is to structure prospective testing rather than to summarize established efficacy.

This explicit framing is intended to support balanced interpretation and to distinguish between areas ready for translational uptake and those requiring further rigorous evaluation.

## 8. A Stage-Dependent Therapeutic Concept

**Figure 4:**
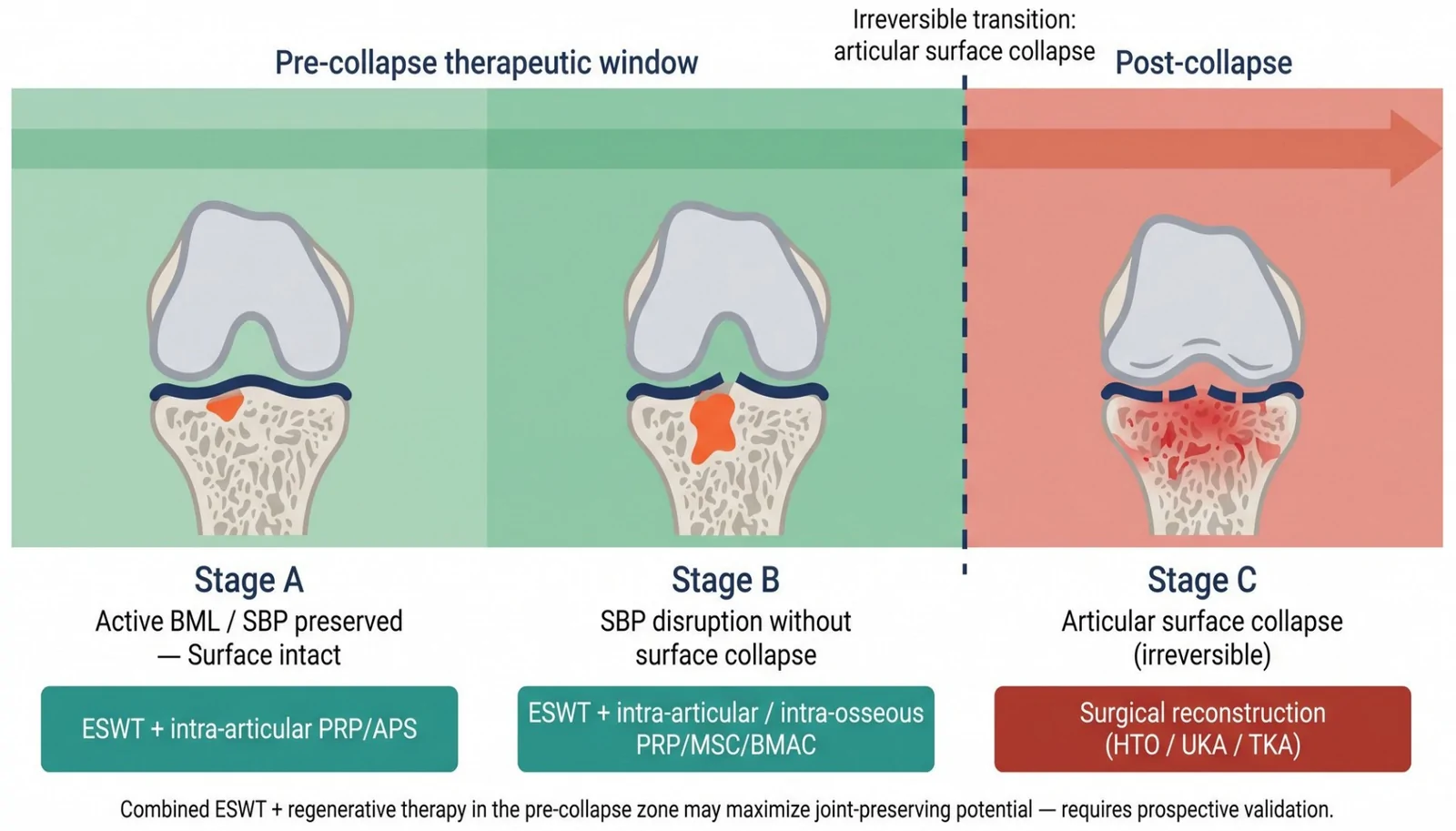
Conceptual pre-collapse therapeutic window. Three stages of disease progression are shown along the horizontal axis. Stage A represents biologically active but structurally preserved subchondral bone (active BML, SBP intact); Stage B represents SBP compromise or discontinuity (Stages 2–3 of Table 1) without articular surface collapse; Stage C represents articular surface collapse. Stages A and B constitute the pre-collapse therapeutic window, in which combined ESWT and regenerative therapy (PRP/APS/MSC/BMAC, intra-articular with or without intra-osseous delivery) is proposed to provide maximal joint-preserving benefit. Stage C is post-collapse and typically warrants surgical reconstruction (HTO/UKA/TKA). Stage definitions are consistent with the text in Section 8. This framework is hypothesis-generating and requires prospective validation. Abbreviations: ESWT, extracorporeal shock wave therapy; PRP, platelet-rich plasma; APS, autologous protein solution; MSC, mesenchymal stromal cell; BMAC, bone marrow aspirate concentrate; SBP, subchondral bone plate; BML, bone marrow lesion; HTO, high tibial osteotomy; UKA, unicompartmental knee arthroplasty; TKA, total knee arthroplasty. The horizontal arrow indicates the direction of disease progression from the pre-collapse stages (A and B) toward the irreversible post-collapse stage (C).

Integrating the preceding sections, we propose a stage-dependent conceptual framework for combined ESWT and regenerative therapy in knee OA (Figure 4). This framework treats stages as biologically continuous, and does not constitute a formal classification system. We state at the outset that the assignment of specific combination strategies to specific stages is an extrapolation from single-modality evidence and mechanistic rationale (Section 7.3); direct evidence for any combination is limited to a retrospective observational series [18], and the framework is intended to organize prospective testing.

Stage A—Biologically active, structurally preserved disease. Symptomatic patients with BMLs, synovitis, and variable cartilage involvement but with a structurally preserved SBP. Intra-articular PRP or APS combined with ESWT is biologically well-suited; symptomatic response can be expected in a substantial proportion of patients [18,23,24,49,50,51,52]. At this stage, the primary therapeutic tasks are to reduce synovial inflammation, stabilize BML activity, and improve the mechanical and vascular microenvironment before structural deterioration progresses. Patient selection at this stage may benefit from evaluation of BML activity, synovitis burden, and joint mechanical alignment; conservative adjuncts including weight management, exercise therapy, and orthoses should continue in parallel [1,2]. The rationale for matching intra-articular PRP or APS to Stage A is compartmental: with a structurally preserved SBP, the dominant treatable biology is intra-articular (synovitis and the joint-fluid milieu), which these products reach directly with their growth-factor and anti-inflammatory cytokine payload—notably interleukin-1 receptor antagonist and soluble TNF receptors in APS [49,50,51,52]—whereas the added invasiveness of intra-osseous access is not justified when the subchondral compartment is intact.

Stage B—SBP compromise or discontinuity without articular surface collapse. Stage B encompasses two structural conditions defined in Section 4.4 and Table 1: microstructural SBP compromise with a continuous plate (Stage 2) and imaging-evident SBP discontinuity (Stage 3); these two terms are used exclusively hereafter, and Figure 4 labels Stage B in this combined sense. Combined intra-articular and intra-osseous delivery of PRP, MSCs (including intra-osseous MSC delivery), or BMAC, together with ESWT-mediated mechanical conditioning, may represent a rational approach [18,39,56,57,58,59,60,61,62,63]. Our clinical observations suggest that this is the stage in which combination therapy—particularly strategies incorporating intra-osseous MSC—provides its greatest incremental value [18]. Conceptually, Stage B is the target of the proposed pre-collapse therapeutic window: the compartment is structurally compromised but not yet geometrically failed, and both cellular and mechanical inputs may still influence the trajectory of repair. The rationale for intra-osseous MSC- or BMAC-based strategies at this stage is likewise anatomical and biological: the therapeutic target lies beneath the SBP, where intra-articular products have limited access (Section 6.1), so the intra-osseous route is required to place the product at the lesion; and the paracrine, immunomodulatory, and osteogenesis-supportive actions of MSC-based products [53,54,55,56] align with the remodeling imbalance that characterizes the subchondral compartment at this stage. This product-to-stage mapping rests on biological rationale and our observational data [18]; no head-to-head randomized comparison between orthobiologic products at any given stage exists, and the mapping is a testable proposal rather than an evidence-based recommendation (Table 4). Prospective identification of patients in Stage B will require imaging protocols capable of resolving focal SBP irregularities and BML activity, together with clinical criteria for articular surface integrity [4,19,26]. In practice, candidate criteria include: for Stage 1 (Stage A), an active BML without fluid-signal foci or micro-cystic change and with a normal SBP contour; for Stage 2, an active BML with focal fluid-signal foci or incipient micro-cystic change beneath a continuous SBP on MRI (supplemented by thin-slice CT where available)—findings that are subtle, of unestablished diagnostic reliability, and proposed here only as candidate criteria pending prospective validation; for Stage 3, discontinuity of the SBP on thin-slice CT or MRI, typically with a communicating cyst-like lesion; and, for Stages 2 and 3, preservation of the bony articular contour. Within Stage B, the incremental benefit of combined ESWT and intra-osseous regenerative therapy is likely greatest in patients in whom microstructural SBP compromise has already occurred but the plate is still continuous, before SBP discontinuity and cyst formation emerge. Notably, the knees in our series [18] were stratified by imaging-evident SBP discontinuity and thus correspond predominantly to Stage 3; the proposition that benefit peaks in Stage 2 is an extrapolation from the mechanical reasoning of Section 4.3 and Section 4.4 and remains to be tested. Once SBP discontinuity becomes evident, the lesion has transitioned toward the irreversible end of the spectrum [30,31], and the same combined therapy is expected to yield progressively smaller structural benefit. Recognizing—and intervening in—this latent, under-recognized phase before SBP discontinuity becomes evident is therefore a central operational implication of the pre-collapse therapeutic window. At the same time, and consistent with the second of the two central messages of this review, radiographically advanced disease—including knees with KL grade 4 changes—does not by itself constitute an unconditional indication for arthroplasty. Because KL grade and structural stage are independent, such knees should be staged by SBP integrity and articular surface status before arthroplasty is assumed to be the only option. When the subchondral compartment retains sufficient viability, a trial of intra-osseous orthobiologic therapy combined with ESWT can provide meaningful symptomatic and, potentially, structural benefit and, in selected patients—as a hypothesis requiring prospective validation—defer or possibly avoid arthroplasty [18,59,60,61,64]. Notably, the MSC-B arm in our series consisted exclusively of KL grade 4 knees, all with imaging-evident SBP discontinuity, and the most favorable trend was observed in the 13 knees without articular surface collapse [18]. Surgical reconstruction therefore remains the treatment of choice when structural failure is extensive and refractory, but should not be regarded as automatically mandated by radiographic grade alone.

Stage C—Articular surface collapse. Once the articular surface has collapsed, mechanical support is lost, and biological reconstitution of macro-scale structure is not achievable with current regenerative tools. Surgical joint reconstruction or arthroplasty often becomes the appropriate consideration [3,11,18]. Biological and mechanotransductive therapies may still have a role for symptomatic management or as adjuncts around surgery, but expectations regarding structural modification should be tempered. Communicating these stage-dependent expectations clearly to patients is essential for informed shared decision-making.

SBP integrity and articular contour constitute the primary structural axis of this framework because they appear to govern prognosis and outcome expectation [18,30,31]; they are not, however, sufficient for treatment selection on their own. Within any stage, selection and co-management should account for the whole-joint context: mechanical alignment (varus or valgus deformity concentrates load on the affected compartment, may warrant orthoses or consideration of osteotomy alongside—or instead of—biological therapy, and uncorrected malalignment is expected to attenuate any biological benefit [3,10,11,12,19]); synovitis burden (a rationale for selecting APS at Stage A [52]); meniscal status, particularly root tears and extrusion, which independently drive progression and may require separate management [12,19]; and systemic factors such as body mass index and metabolic health, addressed through weight management and exercise as continuing co-interventions [1,2]. The framework therefore presupposes concurrent guideline-based conservative management of these whole-joint and systemic factors.

This concept is intentionally kept qualitative. It is not a validated classification system but a mechanistic and clinical heuristic intended to organize thinking about patient selection, outcome expectation, and trial stratification. Because transitions between stages are continuous rather than dichotomous, prospective evaluation using standardized outcome measures and imaging biomarkers of biological activity will be essential. The framework is intentionally compatible with existing MRI-based OA scoring systems and does not require adoption of any new grading nomenclature [26].

## 9. Limitations and Safety Considerations

The proposals in this review must be weighed against several categories of limitation and risk.

Procedural safety of intra-osseous delivery. Intra-osseous injection entails procedural pain, bleeding, and infection risk, and carries a theoretical risk of iatrogenic subchondral fracture, particularly in osteopenic bone; needle placement close to a structurally compromised SBP demands careful trajectory planning. Reported safety in the available series has been acceptable [18,39,59,60,61,62,63,64], but these series are small, and rare complications cannot be excluded. Imaging guidance (fluoroscopic or ultrasound) and operator experience are prerequisites, and outcomes are likely operator-dependent; standardized training and technique description are needed before wider adoption.

Safety of ESWT over compromised subchondral bone. Focused ESWT applied over a discontinuous SBP or a cyst-like lesion could, in principle, accelerate rather than arrest structural failure. No such signal has emerged in the available series [18,35,36,37,38,42,43,44], but these were not designed to detect it. We therefore recommend that subchondral structural staging (Table 1) precede ESWT prescription, that energy titration err conservatively in Stage 3, and that structural imaging be repeated during treatment in structurally advanced knees. The safety of repeated ESWT sessions over months to years, as used in our practice, has not been formally established and warrants dedicated surveillance.

Product variability and regulatory considerations. MSC-based products vary in tissue source, expansion status, dose, and characterization, and manufacturing variability directly affects both efficacy and safety; PRP varies in platelet dose and leukocyte content [49,50,51,52,53,54,55,56,57,58]. Cell-based interventions are subject to jurisdiction-specific regulatory frameworks—in Japan, the Act on the Safety of Regenerative Medicine—and clinical use outside trials should occur within such frameworks, with prospective registries capturing adverse events.

Limitations of the evidence base and of this review. Direct evidence for the combination concept is retrospective and observational [18], with the confounders discussed in Section 7.2; the stage framework, including Stage 2, is unvalidated; and, as a narrative review without a pre-registered protocol or formal risk-of-bias assessment (Section 1), this article is itself subject to selection bias. These limitations collectively define the framework as hypothesis-generating and underscore the primacy of the prospective studies outlined in Section 10.

## 10. Future Research Directions

Several priorities emerge from the preceding synthesis, spanning clinical trial design, biomarker development, treatment optimization, and integration with hard-tissue bioengineering. Two priority questions, corresponding to the two central messages of this review, should be embedded in these prospective designs: (i) whether subchondral-directed combined therapy delivered during the reversible or under-recognized phase (Stages 1–2) can prevent progression to imaging-overt SBP discontinuity and to articular surface collapse, and (ii) whether, in patients with radiographically advanced OA including KL grade 4 disease, the two-modality combination of intra-osseous orthobiologics and ESWT—used together—can produce clinically meaningful and durable joint preservation and reduce the rate of conversion to arthroplasty compared with standard care.

Prospective, controlled clinical trials. Adequately powered randomized studies are needed to test whether combined ESWT and intra-articular ± intra-osseous PRP/APS/MSC therapy (including intra-osseous MSC delivery) improves symptomatic and structural outcomes in defined patient strata. Trials should pre-specify subchondral structural status and BML activity as stratification variables and should use standardized outcome measures including KOOS, WOMAC, imaging-based structural progression, and patient-reported time-to-arthroplasty [4,18,26]. Adaptive and platform trial designs may be particularly efficient for evaluating multiple combination strategies against a common backbone.

Biomarker-based stratification. Trial cohorts should be stratified using MRI-derived indicators of subchondral biological activity (BML size, MOAKS-type scoring, SBP contour) combined with clinical and functional measures [4,26]. Molecular and imaging biomarkers of bone remodeling (CTX-I, PINP), cartilage turnover (uCTX-II, COMP), synovial inflammation, and neurotrophic activity (e.g., NGF) may complement current imaging in defining biologically active phenotypes and in monitoring treatment response [7,8,9,20]. Integration of these biomarkers into stratified trial designs could accelerate identification of responder subgroups.

Treatment timing. The concept of a pre-collapse therapeutic window implies that earlier intervention may be more effective. Prospective studies should examine time from BML detection to intervention as an outcome-modifying variable, and should assess whether repeated cycles of combined therapy are required to maintain benefit. Whether earlier intervention in Stage A can prevent progression to Stage B, and whether aggressive intervention in Stage B can reduce transition to Stage C, represent central open questions.

ESWT parameter optimization. Energy flux density, pulse count, session number, focused versus radial modality, and anatomic targeting relative to BML location warrant systematic optimization for subchondral bone applications [23,24]. Dose-finding trials with imaging-based response endpoints could substantially improve reproducibility across centers, and standardization of treatment protocols would facilitate meaningful comparison and pooled analyses.

Intra-osseous regenerative therapy (intra-osseous PRP, MSC, and BMAC). Standardization of intra-osseous PRP, MSC, and BMAC delivery—needle placement, volume, cell source and dose, product characterization, imaging guidance, and delivery depth—is essential. Dose–response relationships for intra-osseous MSC, including cell dose, culture conditions, and delivery vehicles, represent a priority for future investigation [18,39,56,57,58,59,60,61,62,63]. Registry-based studies may complement randomized trials by capturing real-world variability in technique and outcome.

Hard-tissue bioengineering integration. Concepts from hard-tissue engineering—including controlled-release delivery vehicles, scaffold-supported orthobiologics, mechanically instructive constructs, and biofabrication or bioink technologies—may in the future be combined with mechanotransductive stimulation such as ESWT to further enhance subchondral repair [15,16,17]. Injectable hydrogels loaded with MSCs or growth factors, extrusion-based bioinks for osteochondral gradient constructs, and 3D-printed calcium phosphate or bioactive glass carriers represent particularly promising translational directions [15,16,17]. Approaches that combine intra-osseous MSCs with controlled-release carriers, and that use ESWT to activate mechanotransductive pathways within engineered constructs, appear especially well aligned with the scope of the present Special Issue on Hard Tissue Engineering and 3D Bioprinting and with the broader ambition of moving from generic orthobiologic injections to engineered, site-specific interventions.

DMOAD and regenerative trial design. Emerging DMOAD candidates and regenerative therapies should be evaluated using stratification schemes that recognize the osteochondral unit as the therapeutic target [1,2,3,11]. Trials that ignore subchondral structural status risk diluting the effect of biologically effective interventions across mechanically heterogeneous populations, potentially generating misleadingly negative results.

## 11. Conclusions

Knee OA is not a disease confined to cartilage. It is a whole-joint disorder in which the subchondral bone plays biologically active and mechanically decisive roles. BMLs, subchondral angiogenesis, altered remodeling, and structural failure of the SBP are central features of disease progression, and each is a candidate target for combined mechanical and biological therapy. Regenerative medicine must engage this compartment directly to move beyond incremental symptomatic benefit.

Beyond cartilage regeneration, the next chapter of biological joint preservation in knee OA hinges on two clinical imperatives that this review has developed throughout. First, BMLs are potentially reversible early but progress to irreversible SBP discontinuity, subchondral cyst formation, and articular surface collapse if left untreated—so subchondral-directed treatment should be delivered before SBP discontinuity and cyst formation become imaging-overt and the lesion becomes irreversible. Second, advanced radiographic disease, including KL grade 4 knees, is not by itself an unconditional indication for arthroplasty: the combination of intra-osseous orthobiologics and ESWT—understood as the two modalities acting together, not orthobiologics alone—may, in selected patients and as a hypothesis requiring prospective validation, provide meaningful joint preservation and defer—or in some cases avoid—surgery; symptomatic improvement, structural preservation, and arthroplasty avoidance are distinct outcomes, and evidence for the latter remains insufficient. Rigorous prospective validation of stage-dependent combined mechanical and biological interventions—organized around these two clinical questions—is the appropriate next step, and one that invites closer collaboration between clinical orthopedics, regenerative medicine, and hard-tissue bioengineering to reshape how knee OA is diagnosed, staged, and treated.

## Figures and Tables

**Table 1:** Working imaging definitions of the four-stage subchondral continuum and its mapping to the therapeutic concept of Section 8.

Stage	SBP Continuity	Subchondral Cyst-Like Lesions	Bony Articular Contour	Overlying Cartilage (Typical)	Reversibility/Defining Transition
Stage 1—active BML (Stage A)	Continuous, normal contour	Absent	Preserved	Full thickness	Potentially reversible
Stage 2—microstructural SBP compromise; hypothesized latent phase (core of Stage B)	Continuous on routine imaging; compromise inferred, not directly diagnosable	Absent (at most sub-threshold fluid-signal foci without visible joint communication)	Preserved	Possible early thinning	Elevated risk of irreversible progression; transition 2 → 3 = loss of SBP continuity on MRI/thin-slice CT
Stage 3—SBP discontinuity (late Stage B)	Interrupted on routine MRI or thin-slice CT	Typically present; may communicate with joint	Preserved	Substantially thinned or focally lost	Markedly reduced; transition 3 → 4 = collapse of bony articular contour
Stage 4—articular surface collapse (Stage C)	Interrupted	Often present	Collapsed	Extensively lost	Irreversible

These are working definitions for a conceptual, hypothesis-generating framework and do not constitute a validated classification. Stage 2 is a hypothesized latent phase inferred from histological studies [4,26,27,28,29] and cannot currently be diagnosed objectively on routine imaging; prospective imaging validation is required. The stages are compatible with, and can be operationalized using, MOAKS-type semi-quantitative feature scoring [26]. BML, bone marrow lesion; SBP, subchondral bone plate; MRI, magnetic resonance imaging; CT, computed tomography; MOAKS, MRI Osteoarthritis Knee Score.

**Table 2:** Clinical outcomes across four treatment strategies stratified by articular surface status (quantitative KOOS values, mean ± SD; unit of analysis: knee; adapted from Nakasato et al., 2025 [18]). “SBP disruption”/“SBP tear” in [18] denotes imaging-evident interruption of the plate, corresponding to Stage 3 in Table 1.

Treatment Arm (Cases; KL Grades)	Stratum (Knees)	Baseline	3 M	6 M	IV (Pre–6 M)	Within-/Between-Group *p*
ESWT alone—focused shock waves to BML region; 40 patients/41 knees (KL2 8, KL3 20, KL4 13); 6-month data available for 21 knees	Collapse absent (*n* = 18)	53.8 ± 21.8	66.4 ± 21.7	72.5 ± 16.4	18.6 ± 13.9	*p* < 0.05 (pre–6 M)
	Collapse present (*n* = 3)	55.5 ± 10.6	56.0 ± 10.2	59.9 ± 12.0	4.4 ± 6.5	n.s.
APS + ESWT—intra-articular APS + ESWT; 47/54 (KL2 9, KL3 15, KL4 30)	Collapse absent (*n* = 46)	47.3 ± 16.9	61.5 ± 20.7	64.5 ± 20.8	17.2 ± 15.4	*p* < 0.001 (pre–6 M)
	Collapse present (*n* = 8)	34.8 ± 8.8	47.0 ± 12.1	47.8 ± 13.1	13.0 ± 17.4	between-strata n.s.
MSC-A + ESWT—intra-articular MSC + ESWT; 12/16 (KL2 1, KL3 4, KL4 11)	Collapse absent (*n* = 12)	55.2 ± 19.2	69.0 ± 18.7	68.7 ± 15.0	13.5 ± 11.4	between-strata *p* = 0.014 at 6 M
	Collapse present (*n* = 4)	42.6 ± 12.8	47.5 ± 12.8	39.3 ± 9.8	−3.3 ± 5.2	
MSC-B + ESWT—intra-articular + intra-osseous MSC + ESWT; 18/19 (KL4 only; all knees with SBP discontinuity)	Collapse absent (*n* = 13)	49.1 ± 18.1	62.1 ± 18.8	64.9 ± 18.9	15.8 ± 8.6	*p* < 0.05 (pre–6 M)
	Collapse present (*n* = 6)	49.8 ± 14.1	47.0 ± 10.4	45.3 ± 13.3	−4.4 ± 7.7	n.s.

APS, autologous protein solution; BML, bone marrow lesion; ESWT, extracorporeal shock wave therapy; KL, Kellgren–Lawrence; KOOS, Knee Injury and Osteoarthritis Outcome Score; MSC, mesenchymal stromal cell; SBP, subchondral bone plate; IV, improvement value (change from baseline); n.s., not significant. SBP discontinuity/articular surface collapse counts in the full cohorts of [18]: ESWT alone—13/41 and 6/41; APS + ESWT—30/54 and 8/54; MSC-A + ESWT—14/16 and 4/16; MSC-B + ESWT—19/19 and 6/19. In the full 3-month ESWT-alone cohort (*n* = 41 knees), IV (pre–3 M) was 20.9 ± 16.1 in collapse-absent knees (*n* = 35) vs. 1.3 ± 7.2 in collapse-present knees (*n* = 6); between-strata *p* = 0.0058. In the subgroup with SBP discontinuity but no collapse (original Table 3 of [18]), mean KOOS scores were: ESWT alone (*n* = 8) 50.0 → 59.0 → 65.8 (IV pre–6 M 15.8 ± 16.0); APS + ESWT (*n* = 22) 44.6 → 54.4 → 57.6 (13.0 ± 14.7); MSC-A + ESWT (*n* = 11; KL3 4, KL4 7) 48.8 → 66.2 → 65.6 (16.8 ± 8.9); MSC-B + ESWT (*n* = 19) 49.1 → 62.1 → 64.9 (15.8 ± 8.6); one-way ANOVA with Bonferroni correction showed no significant between-group differences in IV (pre–6 M). Data derive from a retrospective, non-randomized, single-center observational study in which treatment allocation followed clinical characteristics; groups differ in size, KL distribution, and baseline scores, and no comparative-effectiveness inference should be drawn (see Section 7.2). Statistics not reported in [18] are left blank.

## Data Availability

No new data were created or analyzed in this study. Data sharing is not applicable to this article. All data discussed in this narrative review are derived from previously published studies cited in the article.
